# Sclera-related gene polymorphisms in high myopia

**Published:** 2009-08-20

**Authors:** Hui-Ju Lin, Lei Wan, Yuhsin Tsai, Su-Ching Liu, Wen-Chi Chen, Shih-Wei Tsai, Fuu-Jen Tsai

**Affiliations:** 1 Department of Ophthalmology, China Medical University Hospital, Taichung, Taiwan; 2 Department of Medical Genetics, China Medical University Hospital, Taichung, Taiwan; 3 School of Chinese Medicine, College of Chinese Medicine, China Medical University, Taichung, Taiwan; 4 Department of Biotechnology, Asia University, Taichung, Taiwan; 5 Graduate Institute of Integrated Medicine, College of Chinese Medicine, China Medical University, Taichung, Taiwan; 6 Institute of Environmental Health, College of Public Health, National Taiwan University, Taipei, Taiwan

## Abstract

**Purpose:**

Transforming growth factor-β2 (TGF-β2), basic fibroblast growth factor (bFGF), and fibromodulin (FMOD) are important extracellular matrix components of the sclera and have been shown to be associated with the development of high myopia. Our aim was to examine the association between myopia and the polymorphisms within *TGF-β2*, *bFGF*, and *FMOD*.

**Methods:**

The study group comprised of patients (n=195; age range: 17−24 years) with a spherical equivalent of −6.5 diopters (D) or a more negative refractive error. The control group comprised of individuals (n=94; age range: 17−25 years) with a spherical equivalent ranging from −0.5 D to +1.0 D. The subjects with astigmatism over –0.75 D were excluded from the study. High resolution melting (HRM) genotyping and restriction fragment length polymorphism (RFLP) genotyping were used to detect single nucleotide polymorphisms (SNPs). The polymorphisms detected were *TGF-β2* (rs7550232 and rs991967), *bFGF* (rs308395 and rs41348645), and *FMOD* (rs7543418). Moreover, a stepwise logistic regression procedure was used to detect which of the significant SNPs contributed to the main effects of myopia development.

**Results:**

There were significant differences in the frequency of the A allele and A/A genotype in *TGF-β2 *(rs7550232; p=0.0178 and 0.03, respectively). Moreover, the haplotype distribution of haplotype 2 (Ht2; A/A) of *TGF-β2* differed significantly between the two groups (p=0.014). The results of the stepwise logistic regression procedure revealed that *TGF-β2* (rs7550232) contributed significantly to the development of high myopia.

**Conclusions:**

TGF-β2 is an important structure of sclera and might contribute to the formation of myopia. *TGF-β2 *(rs7550232) polymorphisms, A allele and A/A genotype, had a protective role against the development of high myopia.

## Introduction

The prevalence of myopia ranges from 20% to 30% in North American, European, and Australian populations [1] and is as high as 90% in Asian populations [2,3]. Low to moderate degrees of myopia present a relatively minor inconvenience as the symptoms are easily corrected using spectacles or contact lenses. High degrees of myopia, while also correctable using these optical approaches, are of major concern because of the increased risk of myopia-related pathology such as chorioretinal degeneration and retinal detachment [4]. The ocular pathology associated with high myopia is among the leading causes of registered blindness in the developed world [5].

Many studies have suggested that myopia is a complex disease with multiple causes, including the interaction of multiple genes with environmental stimuli [6]. Therefore, it is necessary to take both genes and environment into account to understand myopia [6]. As for pathogenesis of high myopia, studies of high myopia in animal models have demonstrated that increasing eye size facilitated by remodeling of the sclera was one of the most important etiologies in the progression of myopia [7]. The sclera consists of fibrous connective tissue comprised largely of heterologous collagen fibrils, which are in turn composed mainly of type I collagen with small amounts of other fibrillar and fibril-associated collagens [8]. An important candidate in the search for factors involved in scleral remodeling in myopia is transforming growth factor-β (TGF-β). Transforming growth factor-β is known to initiate myofibroblast formation and fibrosis [9]. Although five members of the TGF-β family have currently been identified, only TGF-β isoforms 1, 2, and 3 have been detected in eyes and TGF-β2 is the predominant form [9,10].

In addition to TGF-β, basic fibroblast growth factors (bFGFs) are also involved in the remodeling of sclera. bFGFs with their receptors (FGFRs) and signaling cascades are involved in a diverse range of cellular processes including proliferation, apoptosis, cell survival, chemotaxis, cell adhesion, motility, and differentiation. bFGF-2 is a potent mitogen for fibroblasts and myofibroblasts [11]. Moreover, the major extracellular matrix components of the fibrous mammalian sclera are collagen types I and III and the small leucine rich proteoglycans (SLRPs), which include decorin, biglycan, lumican, and fibromodulin (FMOD) [12]. There is considerable evidence that FMOD is involved in regulating the formation of the network of collagen fibrils that makes up the extracellular matrix [13]. Among the polymorphisms, rs7550232 (*TGF-β2*) and rs308395 (*bFGF*) are found in the promoter region and rs991967 (*TGF-β2*) and rs41348645**(*bFGF*) are found in the 3′-UTR region. Polymorphisms in 5′-UTR and 3′-UTR might have effects on the translation and stability of the genes. We selected these polymorphisms as candidates in this study.

## Methods

### Patients

From February to November 2004, we measured the refractive error in 3,000 volunteers. All of the participants were medical students, unrelated and Taiwan-born Han Chinese (Table 1). The included subjects had a visual acuity with distance correction of 0.2 logMAR (logarithm of the minimal angle of resolution; 20/32) or better. The included subjects did not have any systemic diseases. They also did not receive ocular surgery before. Refractive error was measured in diopters (D) and determined by the mean spherical equivalent (SE) of the two eyes of each individual after administering one drop of cycloplegic drug (1% mydricyl; Alcon, Berlin, Germany). Individuals with −6.5 D or a more negative refractive error (both eyes) were included in the study group, and those with myopia between −0.5 D and +1.0 D (both eyes) were included in the control group. Patients with astigmatism with a refractive error more negative than -0.75 D were excluded from the study. Other volunteers did not meet the criteria to be included in either the study or control groups, and they were excluded from our study. Our study was reviewed by the ethics committee, and informed consent was obtained from all patients and control subjects. The study was performed according to the tenets of the Declaration of Helsinki for research involving human subjects. Comprehensive ophthalmic examinations and blood collection were performed. As with all data collection procedures, auto-refraction (Auto-refractor/auto-keratometer; ARK 700A; Nikon, Tokyo, Japan) was taken on both eyes by experienced optometrists who were trained and certified on study protocols. Refractive data, sphere(s), negative cylinder©, and axis measurements were analyzed by calculating refractive error with SE.

**Table 1:** Characteristics of the study subjects.

**Characteristics**	**Control (n=94)**	**Cases (n=195)**	**All subjects (n=289)**
Age, mean (SD), yr	18 (2.9)	18 (3.4)	18 (3.2)
Female, Number (%)	33 (35.1%)	70 (35.9%)	103 (35.6%)
SE, mean (SD), D	0.02 (0.32)	−8.83 (2.5)	−4.5 (4.8)
AXL, mean (SD), mm	23.56 (0.78)	26.8 (1.8)	24.8 (2.5)
CD, mean (SD), D	43.5 (0.9)	44.2 (1.9)	43.8 (1.5)
ACD, mean (SD), mm	3.56 (0.25)	3.88 (0.33)	3.62 (0.3)
LT, mean (SD), mm	3.8 (0.65)	4.0 (0.58)	3.9 (0.6)

SE: spherical equivalent, AXL: axial length, CD: cornea diopter, ACD: anterior chamber depth, LT: lens thickness.

### Genotype determinations

The single nucleotide polymorphisms (SNPs) were selected based on three criteria. (1) The selected SNP had a frequency in the CHB (Han Chinese in Beijing, China) or JPN(Japanese) population as documented in the HapMap project, and the heterozygosity should be more than 10%. (2) The selected SNP should be in the region of the promoter, 5′-UTR, exons, and 3′-UTR of the gene. We excluded any intron SNP because it is hard to predict the outcome of the polymorphism. (3) In exons, only non-synonymous SNPs were selected. Based on these criteria, only a few SNPs could be selected. For *FMOD*, the selected SNP was based on sequencing experiments. The exons of *FMOD* were sequenced among 50 randomly selected individuals to reveal the SNPs in *FMOD*. Only one SNP was detected. As for selecting haplotype, HapMap genotypes were analysed within Haploview and tag SNPs were selected using tagger. Four tag SNPs were selected for each gene with r^2^≥0.80 to capture 80% of genotype information in the region. The average tag SNP was with r^2^=0.901.

**Figure 1:**
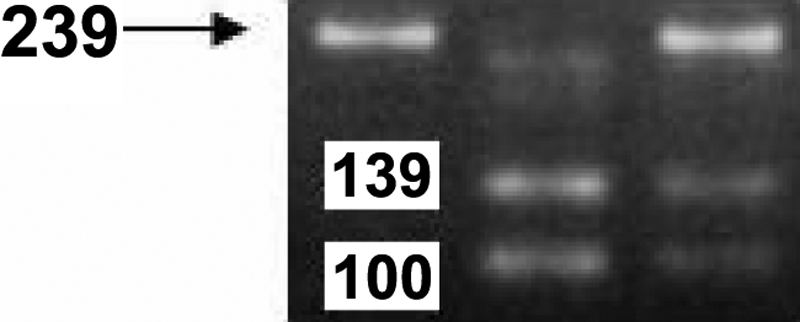
The PCR product of *TGF-β2* (rs991967) genetic polymorphism The PCR product of *TGFβ2*-rs991967 was digested with HpyCH4III. The “*A*” allele was 239 bp and the “*C*” allele was 100 bp + 139 bp.

**Figure 2:**
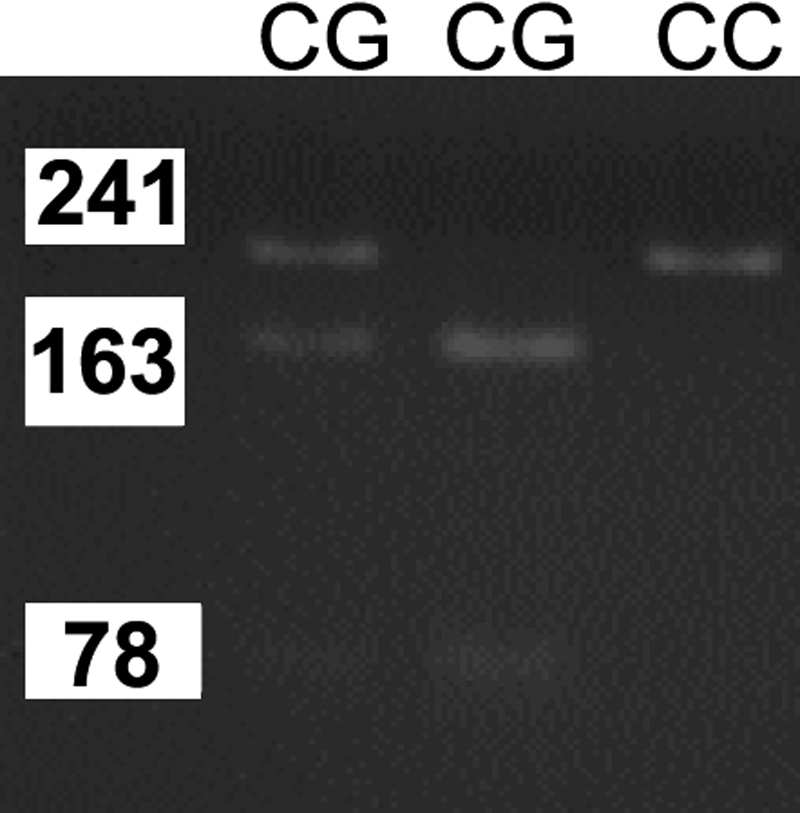
The PCR product of *bFGF *(rs308395) polymorphism. The PCR product of* βFGF2*-rs308395 polymorphism was digested with BsrI. The “*C*” allele was 241 bp and the “*G*” allele was 163 bp + 78 bp.

**Figure 3:**
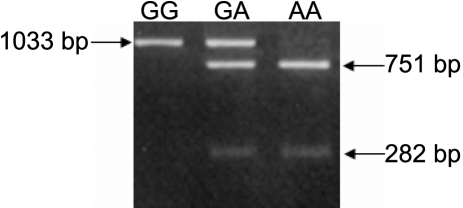
The PCR product of* FMOD* exon 2 codon 79 genetic polymorphism. The PCR product of* FMOD* exon 2 codon 79 genetic polymorphism was digested with BsmI. The “*G*” allele was 1,033 bp and the “*A*” allele was 751 bp + 282 bp.

In this study, we used high resolution melting (HRM) genotyping and restriction fragment length polymorphism (RFLP) genotyping to detect the SNPs. Genomic DNA was extracted from whole blood samples after a standard protocol of digestion by proteinase K and purification with phenol-chloroform. RFLP was used to detect genotypes of *TGF-b2 *(rs991967), *bFGF* (rs308395), and *FMOD* (rs7543148). The amplification protocol and restriction enzymes used to determine the genotype are listed in Table 2. Preventive contamination measures were taken by including a polymerase chain reaction (PCR) reaction mixture without DNA (negative control) in each run of amplification. The DNA fragments were separated by horizontal electrophoresis on 3% agarose gels, stained with ethidium bromide, and photographed under ultraviolet lights. Data were analyzed by ABI prism GeneMapper Version 3.0 software (Applied Biosystems Co, Foster City, CA). The PCR product of *TGF-β2* (rs991967) was digested with HpyCH4III (New England Biolabs, Mississauga, Ontario, Canada). The A allele size was 239 bp, and the C allele size was 100 bp+139 bp as shown in Figure 1. The PCR product of *bFGF* (rs308395) polymorphism was digested with BsrI (New England Biolabs). The C allele size was 241 bp, and the G allele size was 163 bp+78 bp as shown in Figure 2. The PCR product of the *FMOD* (rs7543148) genetic polymorphism was digested with BsmI (New England Biolabs). The G allele size was 1,033 bp, and the A allele size was 751 bp+282 bp as shown in Figure 3.

**Table 2:** Conditions of PCR and HRM. The primer pairs, condition, product size, and restriction enzyme of *TGF-β2* (rs991967), *bFGF* (rs308395) in the PCR-RFLP and *TGF-β2 *(rs7550232) and *bFGF *(rs41348645) in the reaction of HRM reaction.

**Sequence Polymorphism (rs number)**	**Primer**	**PCR Product size (bp)**	**PCR conditions (annealing temperature)**	**Restriction enzyme site**	**Alleles**	**DNA Fragment size (bp)**
*TGF-β2*rs991967	F: 5′-TGACCGAGAAAGTCTGCATT-3′	239	55 °C	HpyCH4III	A	239
	R: 5′-AAGGTCTGAAGTTTGACCAGTACA-3′				C	100+139
*bFGF*rs308395	F: 5′-GCATGGCCTTTTGAAACCTA-3′	241	55 °C	BsrI	C	241
	R: 5′-CAGCGTCTCACACACTGAGG-3′				G	163+78
*FMOD*rs7543148	F: 5′-GCTGG CTTGC TCTGT TCTCT-3′	1033	55 °C	BsmI	G	1033
	R: 5′-GCCAA GGTCT CACCA TTGAT-3′				A	751+282
**Sequence Polymorphism (rs number)**	**Primer**	**DNA concentration**	**Mg concentration**	**PCR conditions (annealing temperature)**		**Alleles**
*TGF-β2*rs7550232	F: 5′-AACGGGAGACTTGATTGTCCT-3′	7.8 ng	2 mM	touchdown 60/53 °C		A
	R: 5′-CGAACCGTTGAGGGAGTGT-3′					C
*bFGF*rs41348645	F: 5′-ACCATAGACTGTCTTACCCA-3′	7.8 ng	2 mM	touchdown 60/53 °C		A
	R: 5′-CAATTGTAAGGGAAGTCAGC-3					G

In this study, HRM genotyping was used to detect the SNPs of *TGF-β2 *(rs7550232) and *bFGF *(rs41348645). Real-time PCR amplification of the *16S rRNA* gene was performed in a LightCycler® 480 High Resolution Melting Master (Roche Diagnostics Co, Mannheim, Germany) with the primer pairs listed in Table 2. Briefly, genomic DNA (50 ng) was added to a 10 µl reaction mixture containing 0.5 µmol/l and 2 mmol/l MgCl_2_ of each primer. Cycling conditions are listed in Table 2. We also compared the haplotypes of these polymorphisms in their susceptibility to high myopia. Haplotypes were inferred from unphased genotype data using the Bayesian statistical method available in the software program, Phase 2.1. All five SNPs were analyzed with the Phase 2.1 software [14]. We defined haplotype 1 (Ht 1) to haplotype 4 (Ht 4) as the alternative allelic composition of the *TGF-β2* and *bFGF* polymorphisms. They are defined in Table 3.

**Table 3:** Gene haplotypes of *TGF-β2* rs7550232/rs991967and *βFGF* rs308395/rs41348645.

SNPs	**Haplotype**		**Controls (%)**	**Cases (%)**	**OR (95% CI)**	**p value**
*TGF-β2*rs7550232/rs991967	Ht1	A/C	126 (67.0%)	258 (66.1%)	0.94 (0.65–1.35)	0.736
	Ht2	A/A	30 (16.0%)	96 (24.60%)	1.75 (1.12–2.75)	0.014
	Ht3	C/C	26 (13.8%)	36 (9.20%)	0.64 (0.37–1.09)	0.101
	Ht4	C/A	6 (3.1 %)	0 (0.00%)	-	N#
*bFGF*rs308395/rs41348645	Ht1	C/A	116 (61.7%)	226 (57.9%)	0.88 (0.62–1.24)	0.458
	Ht2	C/G	48 (25.5%)	114 (29.2%)	1.2 (0.81–1.77)	0.360
	Ht3	G/A	24 (12.8%)	32 (8.2%)	0.59 (0.34–1.02)	0.058
	Ht4	G/G	0 (0%)	18 (4.6%)	-	N#

#; The case number was too small and under power for analyzing.

### Statistical analysis

Genotypes were obtained by direct counting with subsequent calculation of allele frequencies. Data were analyzed using the χ^2^ test, and p values were calculated using the Minitab program (Minitab Inc., San Jose, CA). A p value less than 0.05 was considered significant. Adherence to the Hardy–Weinberg equilibrium (HWE) constant was tested using a χ^2^ test with one degree of freedom. The odds ratios (OR) and the corresponding 95% confidence intervals (CI) were calculated with reference to the allele and genotype. Correction for multiple comparisons was performed by Bonferroni correction. For the SNPs that were found to be significant after multiple testing corrections, stepwise logistic regression was used to determine if any of these SNPs could account for the effects of the other positive SNPs. STATA package (version 8.2; Stata Corp., College Station, TX) was used to perform the stepwise logistic regression.

## Results

The volunteers who enrolled in this study all met specific criteria: age ranging from 16 to 25 years (mean age, 18±3.2 years), male-to-female ratio of 1.8–1.0, mean axial length (AXL) of 24.8 mm, and mean spherical equivalent (SE) of 4.5 D. There were no significant differences between the control and cases groups in age, gender, cornea diopter (CD), anterior chamber depth (ACD), and LT (lens thickness; by Zeiss IOL master [Zeiss Inc, Boston, MA]; Table 1). The study group comprised of 195 patients with high myopia, and the control group consisted of 94 control subjects. The *TGF-β2* (rs991967) genotypes were A/A, A/C, and C/C (Figure 1). The genotype and allele distribution of the *TGF-β2* (rs991967) polymorphism did not differ significantly between high myopia patients and normal controls (p=0.437 and p=0.169, , respectively; Table 4). The *bFGF* (rs308395) genotypes were G/G, C/G, and C/C (Figure 2). The genotype and allele distributions of the *bFGF* (rs308395) polymorphism did not differ significantly between high myopia patients and normal controls (p=0.215 and p=1.0, respectively; Table 4). In the *FMOD* (rs7543418) genetic polymorphism, the genotype and allele distributions of the *FMOD* (rs7543148) polymorphism also did not differ significantly between high myopia patients and normal controls (p=0.362 and p=0.129, respectively; Table 4).

**Table 4:** The genotype distribution of polymorphisms between the high myopia group and control subjects.

**SNP**	**Genotype**	**Controls**	**Cases**	**OR (95% CI)**	**p-value/ Cp-value#**	**Allele**	**Controls**	**Cases**	**OR (95% CI)**	**p-value/ Cp-value#**
*TGF-β2*rs7550232	n	94	195			n	188	390		
(S1)	A/A	63 (67%)	160 (82%)	1.00	0.015/0.03	A	156 (83%)	353 (91%)	1.00	0.0089/0.0178
	C/C	1 (1%)	2 (1%)	0.79 (0.07–8.84)		C	32 (17%)	37 (9%)	0.51(0.31–0.85)	
	A/C	30 (32%)	33 (17%)	0.43 (0.24–0.77)						
	HWEc2	1.583	0.042							
*TGF-β2*rs991967	n	94	195		0.2189/0.4378	n	188	390		
(S2)	C/C	61 (65%)	109 (56%)	1.00		C	152 (81%)	290 (74%)	1.00	0.0847/0.1694
	A/A	3 (3%)	14 (7%)	2.48 (0.68–9.04)		A	36 (19%)	100 (26%)	1.41(0.91–2.17)	
	A/C	30 (32%)	72 (37%)	1.30 (0.76–2.21)						
	HWEc2	0.089	0.19							
*bFGF*rs308395	n	94	195		0.1078/0.2156	n	188	390		
(S3)	C/C	70 (74%)	152 (78%)	1.00		C	164 (87%)	341 (87%)	1.00	1.0/1.0
	G/G	0	6 (3%)	-		G	24 (13%)	49 (13%)	0.97 (0.58–1.62)	
	C/G	24 (26%)	37 (19%)	0.69 (0.38–1.24)						
	HWEc2	2.01	3.63							
*bFGF*rs41348645	n	94	195		0.0647/0.1294	n	188	390		
(S4)	G/G	2 (2%)	8 (4%)	1.00		G	48 (26%)	131 (34%)	1.00	0.0479/0.0958
	A/A	48 (51%)	72 (37%)	2.91 (0.59–14.32)		A	140 (74%)	259 (66%)	1.52 (1.03–2.25)	
	A/G	44 (47%)	115 (59%)	1.71 (1.03–2.85)						
	HWEc2	5.01*	20.2*							
*FMOD*rs7543148	n	94	195			n	188	390		
(S5)	G/G	11 (11.7%)	12 (6.1%)	1	0.181/0.362	G	63 (34%)	106 (27.2%)	1	0.129
	A/A	42 (44.6%)	101 (51.8%)	0.44 (0.19–1.08)		A	125 (66%)	284 (72.8%)	0.72(0.45–1.05)	
	A/G	41 (43.6%)	82(42.1%)	0.58 (0.22–1.37)						
	HWEc2	0.042	0.76							

#; Cp-value, p value corrected by Bonferroni correction; HWE χ^2^, Hardy-Weinburg equilibrium χ^2^; SNP, single nucleotide polymorphism; OR, Odds ratio; CI, confidence intervals. The asterisk indicates that the data did not obey Hardy–Weinberg equilibrium

**Figure 4:**
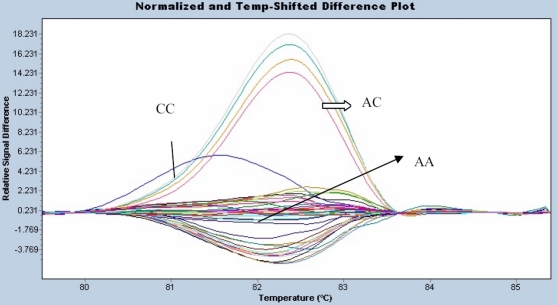
The *TGF-β2* (rs7550232) genetic polymorphism. The *TGFβ2*-rs7550232 genetic polymorphism was studied by HRM and analyzed by LightCycler 480. The *TGFβ2*-rs7550232 genetic polymorphism had three genotypes and they were “CC”, “AC” and “AA”.

**Figure 5:**
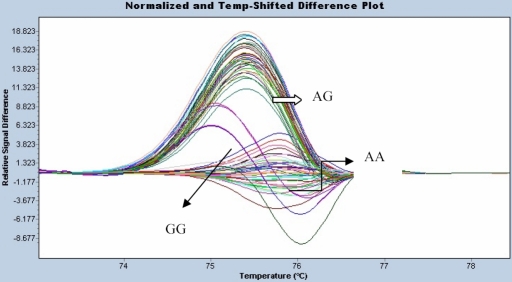
The *bFGF *(rs41348645) genetic polymorphism. The *βFGF2*-rs41348645 genetic polymorphism was studied by HRM and analyzed by LightCycler^®^ 480. The *βFGF2*-rs41348645 genetic polymorphism had three genotypes and they were “GG”, “AG” and “AA”.

HRM was used to detect SNPs of *TGF-β2 *(rs7550232) and *bFGF *(rs41348645)*.* The *TGF-β2* (rs7550232) genetic polymorphism was studied by HRM on a LightCycler 480. The results are shown in Figure 4. The genotype distribution of the *TGF-β2* (rs7550232) polymorphism differed significantly between high myopia patients and normal controls (p=0.03; Table 4). The allelic frequency also differed significantly between the two groups (p=0.0178; Table 4). The genetic polymorphism, *bFGF* (rs41348645), was also studied by HRM. The results are shown in Figure 5. There was no significant difference in genotype and allele distributions of the *bFGF* (rs41348645) polymorphism between high myopia patients and normal controls (p=0.1294 and p=0.0958, respectively; Table 4).

The haplotypes’ distribution of the *TGF-β2* polymorphisms (Ht 1 [A/C], Ht 2 [A/A], and Ht 3 [C/C]) and *bFGF* polymorphisms (Ht1 [C/A], Ht2 [C/G], and Ht3 [G/A]) were compared between the high myopia group and the control group (Table 3). The cases’ numbers of Ht4 in *TGF-β2* polymorphisms and Ht4 of *bFGF* polymorphisms were too small and under powered to analyze (Table 3). Therefore, they were excluded in this analysis. In the analysis of haplotypes, we found that the distribution of Ht 2 (A/A) in *TGF-β2* polymorphisms differed significantly between the two groups (p=0.014; Table 3). However, there was no significant difference in the distribution of Ht 1 and Ht 3 of *TGF-β2* between the two groups (p=0.736 and p=0.101; Table 3). As for the haplotypes’ distribution in *FGF-2*, there was no significant difference in the distribution of all haplotypes of *FGF2* Ht 1 (C/A), Ht 2 (C/G), and Ht 3 (G/A) between the high myopia group and the control group (p=0.458, p=0.360, and p=0.058, respectively; Table 3).

A stepwise logistic regression procedure was used to detect which of the significant SNPs contributed to the development of high myopia (Table 5). By the backward procedure, eliminated S5 (*FMOD* [AGT→AAT]), S4 (*bFGF* [rs41348645], S3 (*bFGF* [rs3083959]), and S2 (*TGF-β2* [rs991967]) from the model did not have any significant effect (p=0.3819, 0.2014, 0.128, and 0.158, respectively; Table 5). In conclusion, the stepwise regression procedure demonstrated that S1 (*TGF-β2* [rs7550232]) significantly contributed the development of high myopia (p=0.004) (Table 5).

**Table 5:** SNPs analyzed by stepwise logistic regression procedure

**Null model**	**Alternative model**	**p value**
S1+S2+S3+S4+S5	S1+S2+S3+S4	0.3819
S1+S2+S3+S4+S5	S1+ S2+S3+S5	0.2014
S1+S2+S3+S4+S5	S1+S2+S4+S5	0.128
S1+S2+S3+S4+S5	S1+S3+S4+S5	0.158
S1+S2+S3+S4+S5	S2+S3+S4+S5	0.004

S1: *TGF-β2*-rs7550232, S2: *TGF-β2*-rs991967, S3: *βFGF*-rs308395, S4: *βFGF*-rs41348645, S5: *FMOD*-rs7543148.

## Discussion

Some genes have been found to be closely related to high myopia such as matrix metalloproteinases [15], paired box 6 [16], myocilin [17], *TGF-β1* [18], Paired box gene 6 (*PAX-6*) [19], and Collagen, type I, alpha 1 (*COL1A1*) [20]. These genes all indicate an evident predisposition for the development of high myopia. However, none of these genes have been found to be solely responsible for the development of myopia in different ethnic groups or to cover all myopia patients [15-21]. The other difficulty in myopic study is the uncertainty of what are the surrounding environmental influences that promote the progression of myopia. Individuals with a higher education have a higher prevalence of myopia than people in the general population, which is why students in a medical school were chosen as candidates in this study. The medical school’s students in this study decreased the bias of environmental influence.

In this study, we found that the distribution of the *TGF-β2* (rs7550232) A/A genotype and A allele differed significantly between the myopia and control groups. The A/A genotype and A allele of *TGF-β2* (rs7550232) had a protective effect against myopia (Table 4). In the analysis of haplotypes, we found that Ht 2 (A/A) of *TGF-β2* was significantly different in the incidence of high myopia. The frequency of the Ht 2 (A/A) of *TGF-β2* was higher in the control group then in the myopia group (p=0.014), indicating that Ht 2 of *TGF-β2* polymorphisms had a protective effect against myopia. In *TGF-β2*, combining the analyses of allelic and haplotype study, we could indicate that the A allele of *TGF-β2* (rs7550232) had a protective effect against high myopia, and its effect would increase when the subjects had the A allele in *TGF-β2* (rs991967; Ht2 [A/A] of *TGF-β2*; Table 3). As for *bFGF,* the frequencies of the haplotypes were all without significant differences between the two groups (Table 3). According to our study, the genetic polymorphisms in *bFGF* did not play any important role in the suffering of high myopia. Besides, the results of stepwise regression procedure demonstrated that S1 (*TGF-β2* [rs7550232]) contributed significantly to the development of myopia (p=0.04) (Table 5). There were no significant effects when S5 (*FMOD* [rs7543148]), S4 (*bFGF* [rs41348645]), S3 (*bFGF* [rs3083959]), and S2 (*TGF-β2* [rs991967]) were eliminated from the model (Table 5). This also reinforced the importance of *TGF-β2* (rs7550232) in the development of high myopia. In the check of the Hardy–Weinberg equilibrium (HWE), all SNPs in this study obeyed HWE except *bFGF *(rs41348645; Table 4). To validate our findings, we repeated the genotyping analysis several times and consistent results were obtained. The errors in genotyping were kept to a minimum. We tried to trace the ancestry background of the controls and patients to check the probability of population stratification. According to the paper published by Pan et al. [22], they mentioned that on the SNP profiles in the major histocompatibility complex (MHC) region (6p21.3), the composition of our population is as follows: 85% are Minnan descendants, 5% are Hakka descendants, and the remaining 10% are mixed population of Minnan, Hakka, and Canton descendants. There was no significant difference in the MHC region among these groups, this indicated that the population in this study was homogeneous and population stratification should not affect this study. Moreover, the deviations from HWE might be a sign of mutation. This disequilibrium seems to also support the association of the polymorphisms in *TGF-β2* with high myopia.

The expression of *TGF-β1* and *TGF-β2* were different in myopia. A study by Hayashi et al. [23] revealed that there was no significant relationship between high myopia and *TGF-β1* among the Japanese [23]. Furthermore, Kusakari et al. [24] reported that *TGF-β1* expression was reduced in the sclera, choroid, and retina during myopia development in an isoform- and time-specific manner. However, it has been shown that the TGF-β2 content increased in the retina, choroid, and sclera during myopia development [25]. In this study, we found that expression of *TGF-β2* was different from the result of *TGF-β1* among the Japanese. The different expressions of *TGF-β2* and *TGF-β1* in myopia could be understood by the different behavior of the two isoforms in sclera during the progression of myopia. Moreover, our other study has found that the CC genotype in the *TGF-β1* codon 10 polymorphism (rs1982073) contributes to the genetic predisposition of high myopia [18]. The SNP we detected (*TGF-β1* [rs1982073]) was not the purpose in the study of Hayashi et al. [23], which is about the relation of *TGF-β1* and myopia in the Japanese. To refine the role of *TGF-β1* in myopia, it is still necessary to study the expression of *TGF-β1* in other populations. Studies on the TGF-β-induced factor gene**(*TGIF*) [26] had revealed that *TGIF* 657 T→G was a useful marker for high myopia. The target genes of these studies were related to *TGF-β1* and not *TGF-β*, which was surveyed in this study. These data may be collected together for mapping high myopia in the future. TGF-β is one of the growth factors released from photoreceptor and retina pigment epithelium layer in the retina [27], TGF-β demonstrated a different broad range of function [28]. A previous study had proposed that the concentration of *TGF-β* mRNA and active form of the TGF-β protein will decrease in the form-deprived myopia eyes compared with the control group [29]. Consequently, TGF-β may mediate the control of ocular axial elongation then influence the progression of high myopia [29]. A recent study about TGF-β revealed that reduced scleral TGF-β is a major contributor to the extracellular matrix remodeling in the myopic eye. In the progression of myopia, remodeling of scleral and producing a larger population of contractile cells are the important process [28]. These findings all revealed that important role of TGF-β in myopia. The function assay of *TGFb2*-rs7550232 and the genetic regulation the SNP is worthy of further investigation. 

Myopia is considered to be a complex and multigenic disease involving several overlapping signaling pathways, each one mediated by a group of distinct genetic profiles. Therefore, it is important to study the polymorphisms involved in the development of myopia to further clarify the relationship between genes and myopia. In this study we prevented the effects of environment by selected the study and control groups from medical school students. Nevertheless, the “environment” effects cannot be completely prevented. Moreover, the incidence of myopia in our country increased in the past 20 years [30]; these all indicated that the effective of environment would not be over-emphasized. Although the results of this study could give us the genetic information of myopia, the prevention of high myopia still needs the efforts of eye doctors and the association of public health.
